# Methylphenidate, Sleep, and the “Stimulant Paradox” in Adult ADHD: A Conceptual Framework for Integrating Chronopharmacotherapy and Coaching

**DOI:** 10.3390/jcm14238494

**Published:** 2025-11-30

**Authors:** Rafał R. Jaeschke, Joanna Z. Sułkowska

**Affiliations:** 1 Section of Affective Disorders, Department of Psychiatry, Jagiellonian University Medical College, 31-501 Kraków, Poland; 2 Students’ Scientific Group of Adult Psychiatry, Faculty of Medicine, Jagiellonian University Medical College, 31-501 Kraków, Poland; joasia.sulkowska@doctoral.uj.edu.pl

**Keywords:** ADHD, methylphenidate, chronopharmacotherapy, sleep, ADHD conceptual, ADHD coaching, ADHD highlighting, ADHD management

## Abstract

This conceptual narrative review explores the intricate dynamics of the ‘stimulant paradox’ in adult ADHD, highlighting the timing-dependent trade-offs associated with stimulant medication. The paradox underscores the complexity of balancing therapeutic benefits against potential side effects, contingent on the timing of administration. Chronopharmacotherapy emerges as a promising framework, integrating circadian and homeostatic processes with pharmacokinetic and pharmacodynamic considerations. This approach suggests that aligning medication timing with individual biological rhythms may optimize outcomes, although the conceptual nature of this integration remains non-prescriptive. ADHD coaching is examined as a complementary strategy, focusing on the development of skill-oriented routines that are sensitive to timing. This adjunctive support may facilitate the practical implementation of chronopharmacotherapy principles, enhancing adherence and effectiveness. Figures and heuristics within the review serve as illustrative tools intended to guide understanding rather than dictate clinical practice. The review identifies critical areas for future research, emphasizing the need for empirical investigation into optimal clock-time windows, phase measures, and chronotype stratification. Additionally, assumptions regarding medication formulations warrant further scrutiny to refine timing-sensitive interventions in adult ADHD management.

## 1. Introduction

Attention-deficit/hyperactivity disorder (ADHD) is a neurodevelopmental disorder that frequently persists into adulthood, contrary to the misconception that it is confined to childhood [1]. In adults, prevalence estimates vary depending on case definition and ascertainment, but population-level prevalence is approximately 2.5–3.5% [2]. The clinical presentation extends beyond inattention, hyperactivity, and impulsivity to encompass significant sleep–wake disturbances [3]. A common pattern is circadian phase delay, characterized by an evening chronotype and postponed dim-light melatonin onset (DLMO), often accompanied by difficulties in initiating sleep and waking at biologically misaligned times; these features reflect underlying chronobiology rather than a mere discordance with “socially expected hours” [3,4,5,6]. The first-person account in Box 1 exemplifies this nightly conflict and its daytime fallout.

The interaction between sleep disturbances and ADHD symptoms forms a bidirectional loop: circadian misalignment exacerbates both conditions, while insufficient or mistimed sleep undermines executive control and emotion regulation. Within this loop, timing is crucial. Some research suggests that the timing of stimulant medication, particularly methylphenidate, can influence treatment outcomes. Aligning treatment with circadian rhythms may enhance efficacy and reduce sleep-related side effects, highlighting the need for a personalized approach to therapy [7,8,9]. ADHD coaching serves as a skill-oriented adjunct that aids in implementing timing-sensitive routines, harmonizing effectively with chronobiological strategies [10,11,12]. The existing evidence base for methylphenidate underscores its relevance in managing ADHD symptoms while considering the complexities of sleep regulation [13,14].

In this review, we introduce the working concept of the “stimulant paradox”—an operational construct used to organize published observations on timing-dependent benefit–risk patterns. Briefly, across adult studies, later-day stimulant exposure is more often associated with insomnia or prolonged sleep-onset latency, whereas anchoring the principal effect within the biological day appears less disruptive [13,14]. Current guidance mentions circadian-aligned dosing only in limited, contextual terms and does not define a new clinical entity; the construct therefore synthesizes existing observations rather than proposing a guideline change. This analysis aims to elucidate the nuanced interplay between chronobiology and pharmacotherapy in ADHD management.

Box 1 **A clinician’s reflection.**

Around 23:30, I am bone-tired, yet sleep is nowhere near; perhaps it is not on the way at all. Had it arrived anyway, it would be a mixed blessing. My mind races with the “may”—that one last patient email, the final slide for tomorrow’s lecture, a quick review of a manuscript. It feels like a non-negotiable effort to mitigate the stress I already anticipate for the next day. By midnight, I am paradoxically wide awake, the screen’s glow replacing the task’s form with a sharp, albeit artificial, focus. I know this is sabotaging my sleep, but the impulse to stop feels like giving up. True drowsiness does not arrive until 02:00 or later. The 07:00 alarm is a physical shock. I will hit snooze until 08:30, starting the day already defeated, trapped in the same unyielding schedule. My cognitive function peaks in the late afternoon, just in time for the cycle to repeat. I am not about ignoring good advice; it is that in those late-night moments, tomorrow’s exhaustion exists only as a piece of data, not a looming reality.

## 2. Methodological Note

### 2.1. Design and Aim

This narrative review was designed to explore the stimulant paradox within the context of chronopharmacotherapy, focusing on the timing of stimulant dosing relative to the biological day. The aim was to synthesize evidence on the interplay between stimulant administration and circadian rhythms, while also considering practical coaching strategies for implementation in clinical settings.

### 2.2. Sources and Eligibility

Sources were selected based on their relevance to the themes of stimulant paradox and chronopharmacotherapy, with a focus on peer-reviewed articles published in English. Eligibility criteria included studies that addressed stimulant pharmacokinetics, dosing schedules, and their effects on circadian rhythms, as well as those offering insights into coaching strategies for behavioral interventions. Exclusion criteria were non-peer-reviewed articles, studies not directly related to the primary themes, and those lacking robust methodological frameworks. A robust methodological framework was defined as including clear research questions, appropriate study designs, well-defined populations, and rigorous data analysis methods.

### 2.3. Study Selection and Appraisal

In line with evidence-based methods, we worked within a pre-existing institutional evidence catalogue on ADHD, stimulant pharmacotherapy, and sleep that comprised 3949 PubMed-indexed records. For the present narrative review, we then performed a focused PubMed search that yielded 137 unique records. Titles and abstracts of these 137 records were screened to identify studies addressing adult ADHD (or closely related clinical populations), stimulant treatment with particular emphasis on methylphenidate, and sleep or circadian outcomes.

Full texts of potentially relevant articles were appraised against predefined inclusion criteria. We operationalized a “robust methodological framework” as studies that: (i) used prospective experimental or observational designs (for example, randomized controlled or crossover trials, or longitudinal cohort studies), (ii) applied explicit diagnostic procedures for ADHD or closely related conditions, (iii) reported quantitative sleep or circadian endpoints, and (iv) were published in peer-reviewed journals. Studies with major methodological limitations (for instance, unclear diagnostic criteria, absence of quantifiable sleep outcomes, or insufficient reporting of key methodological details) were excluded.

A total of 58 publications fulfilled these criteria and formed the empirical core of this narrative review. Additional high-quality narrative reviews, clinical guidelines, and conceptual papers drawn from the broader evidence catalogue were used to contextualize the findings and to illustrate clinical scenarios, but they were not counted as primary empirical studies in the core set.

A two-stage screening process was employed for study selection. Initially, titles and abstracts were reviewed to identify potentially relevant studies. Subsequently, full-text articles were appraised for inclusion based on their methodological rigor and relevance to the review’s themes. An evidence hierarchy was applied, prioritizing studies with strong experimental designs, characterized by randomization, control groups, and blinding. In cases of discordance between studies, a consensus approach was adopted to ensure balanced representation of findings. We screened a total of 137 records, of which 58 met the inclusion criteria.

### 2.4. Coaching and Behavioral Interventions

This section reviews literature on ADHD coaching and related behavioral interventions, focusing on their role as adjuncts to pharmacological treatments in adult ADHD. A thematic narrative approach was employed to select and integrate relevant studies.

We prioritized peer-reviewed publications defining coaching as a structured, skills-based intervention aimed at enhancing self-management, executive functioning, and adherence. The search included both ADHD-specific coaching and health-coaching interventions in chronic conditions, specifically those reporting patient-important outcomes such as improved organization, daily structure, and sleep-related behaviors. This reflects our review’s focus on timing-sensitive treatment strategies.

Given the methodological diversity and limited number of specific studies, we acknowledge the heterogeneity of the evidence base. Health-coaching studies were included to identify transferable mechanisms that could support the integrative proposals discussed below. This approach highlights the potential of coaching to enhance self-regulation within personalized ADHD treatment plans, while maintaining a cautious interpretation of the empirical limitations.

### 2.5. Data Synthesis Strategy

Data from all identified sources were synthesized narratively, precluding quantitative pooling due to the heterogeneity of study designs.

We structured the synthesis around three domains corresponding to the review’s main objectives: (i) pharmacokinetic and pharmacodynamic effects of methylphenidate on sleep and circadian parameters; (ii) timing-sensitive chronopharmacotherapy strategies; (iii) behavioral and coaching-based approaches supporting daily routines and self-regulation.

Within each domain, we summarized study aims and key outcomes, grouping findings by formulation, dosing schedule, and patient population. Particular attention was paid to clinically interpretable patterns consistent across different study designs, while explicitly noting gaps in the evidence base. Consequently, the synthesis is thematically anchored in the totality of the extracted empirical material.

### 2.6. Figure 1 Development

**Figure 1:**
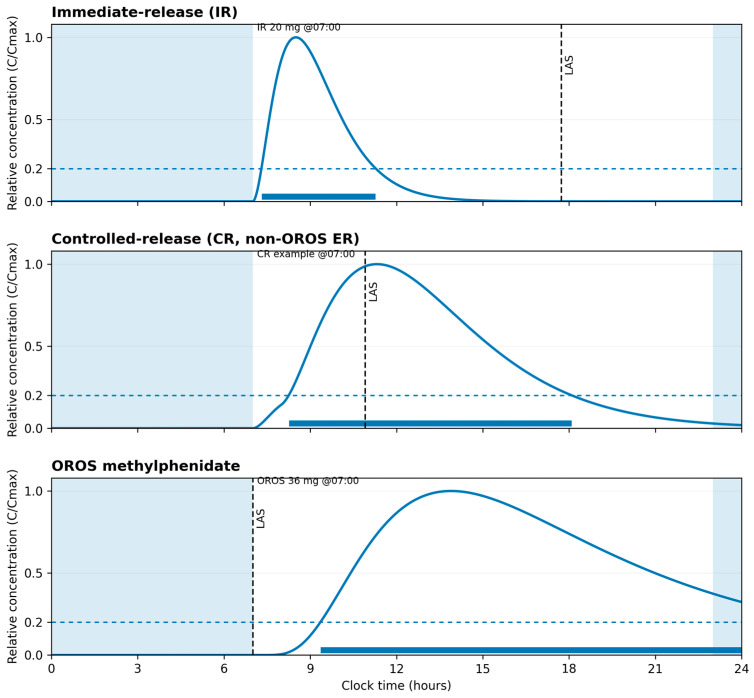
Illustrative methylphenidate pharmacokinetic profiles for immediate-release and extended-release formulations (adapted from Jaeschke et al. [15]).


The pharmacokinetic–pharmacodynamic schema shown in Figure 1 is derived from the dosing profiles and pharmacological characteristics described by Jaeschke et al. [15]. This publication was selected as the primary source because it offers a consolidated description of adult ADHD, mechanisms relevant to sleep regulation, and representative full-day methylphenidate regimens. Using a single, internally consistent reference allowed for coherent visualization of the pharmacokinetic patterns relevant to the present narrative. The figure is intended solely as an explanatory reference for concepts discussed in the manuscript and does not introduce or imply new clinical recommendations.

This schematic shows superimposed concentration–time curves for immediate-release (IR, example dose 20 mg) and extended-release OROS (example dose 36 mg) methylphenidate formulations aligned to clock time and a typical daily schedule. The curves are based on a single-source parameterization from Jaeschke et al. [15] (Tables 4 and 6) and are normalized to C_max_ to illustrate relative time to peak and duration rather than individual measurements or bioequivalence. The three panels depict different dosing scenarios; a shaded band marks usual sleep hours (23:00–07:00), and a dotted horizontal line indicates a relative exposure threshold (0.20·C_max_) used to derive the “latest advisable start” (LAS) within a daytime dosing window (07:00–21:00) with a 60-minute buffer before bedtime. The figure is conceptual and is intended as an explanatory aid to the narrative discussion, not as prescribing guidance or a dosing recommendation. 


### 2.7. Use of Generative AI

Language editing and figure-caption drafting were supported by OpenAI GPT-4o under author supervision, used intermittently across multiple drafting and revision cycles. No clinical recommendations were generated, and no patient-level or identifiable data were provided. All authors verified the accuracy and accept full responsibility for the content.

## 3. Review of Findings

This section integrates the available evidence in three steps. We first examine how methylphenidate can exert paradoxical, context-dependent effects on sleep in adults with ADHD (“the stimulant paradox”), highlighting the tension between therapeutic symptom control and potential sleep disruption. We then explore chronopharmacotherapy as a framework for optimizing treatment by aligning medication timing with biological rhythms. Finally, we consider the human and behavioral layer of care, focusing on how coaching and related interventions may support adherence, implementation of timing-sensitive regimens, and day-to-day self-management.

### 3.1. The Stimulant Paradox

The stimulant paradox describes the seemingly contradictory effects of methylphenidate, a stimulant, on sleep in adults with ADHD. While typically associated with increased alertness, methylphenidate can either alleviate or exacerbate sleep-related issues. In some individuals, it reduces evening restlessness and bedtime procrastination, potentially improving sleep onset, whereas in others, it may contribute to insomnia or reduced total sleep time. This variability is influenced by the interaction between the pharmacokinetic (PK) and pharmacodynamic (PD) properties of methylphenidate and the brain’s circadian (Process C) and sleep homeostatic (Process S) systems (see Box 2) [16,17,18,19].

Box 2 **Process C and process S—a 60-second clinical primer.**

What they regulate (at a glance):

– Process C (circadian)—the endogenous ~24 h timing signal (SCN-driven) entrained by zeitgebers (light, activity). It shapes the phase of sleep–wake propensity, opening a nightly “sleep window” and setting a daytime alertness profile.
– Process S (homeostatic)—the sleep pressure that accumulates as waking time passes and dissipates during sleep; it is reduced by late naps and caffeine and strengthened by consolidated wakefulness.

,

How they interact (clinically intuitive):

– In the evening, S is typically high while C opens the sleep window. Alignment of high S with the appropriate C phase facilitates short sleep-onset latency (SOL).
– Late naps and caffeine lower effective S → patients “miss” the C sleep window and SOL lengthens.
– Circadian delay (evening chronotype) shifts the entire sleep window later; late-evening light and screens can further push C.

,

Implications for stimulant (methylphenidate) timing:

(1) “Effect in the biological day.” Keep the main effect arc within the active C day; let the descending “tail” end before the sleep window to avoid SOL prolongation.
(2) Protect S. Avoid late naps and afternoon/evening caffeine; reduced S compromises sleep initiation regardless of dose.
(3) Fixed wake time + morning light. These anchor C, gradually advancing the sleep window.
(4) Formulation matters. IR allows a narrow, controllable exposure window (easier last-dose cut-off); ER/OROS requires earlier start so the tail does not intrude into the sleep window.
(5) Late “top-ups” carry risk. Afternoon/evening boosters often overlap the sleep window and extend SOL.

,

Fast clinic heuristics (non-directive):

– A–B–C: A—dose after wake, B—keep effect in the biological day, C—enforce a cut-off before wind-down.
– Prioritize clock hygiene and sleep behaviors before dose escalation.
– If SOL worsens, shift dose earlier or change formulation before increasing dose.

,

Note: This educational box is illustrative and non-prescriptive. Foundations of the two-process model [20].

Understanding the interaction between PK/PD and Process C/S is crucial for explaining the dual effects of stimulants on sleep. Process C, driven by circadian rhythms, regulates sleep timing, while Process S, the sleep homeostat, manages sleep pressure accumulation and dissipation [6,21]. Methylphenidate influences these processes by modulating neurotransmitter levels, such as dopamine and norepinephrine, which are involved in wakefulness and attention [22,23]. The timing of administration is pivotal; morning doses may enhance daytime functioning with minimal impact on sleep, whereas late-day doses can delay sleep onset and reduce sleep duration [24,25].

The choice of stimulant formulation—immediate-release (IR), controlled-release (CR), or osmotic-controlled release oral system (OROS)—also affects these outcomes, as each has distinct PK profiles influencing the duration and intensity of drug action [15,26].

### 3.2. Chronopharmacotherapy

Chronopharmacotherapy involves timing medication administration to align with biological rhythms, optimizing therapeutic outcomes and minimizing side effects. This approach is particularly relevant in ADHD treatment, where the timing of stimulant administration significantly impacts efficacy and tolerability. Key principles include understanding circadian phase measures, such as dim light melatonin onset (DLMO), and tailoring medication schedules to individual circadian profiles [3,25].

The formulation-specific arcs of stimulant medications—immediate-release (IR), controlled-release (CR), and osmotic-controlled release oral system (OROS)—are crucial in chronopharmacotherapy. IR formulations provide rapid onset but require multiple daily doses, complicating adherence and increasing the risk of sleep disturbances if taken too late [19,24]. CR and OROS formulations offer extended release, reducing dosing frequency and potentially improving adherence and sleep outcomes by minimizing late-day exposure [16,18].

The concept of a Phase–Exposure Budget is integral, balancing drug exposure timing with the individual’s circadian phase. Aligning stimulant administration with natural peaks in alertness can enhance efficacy while reducing adverse effects on sleep and other circadian-regulated processes. This requires careful consideration of daily schedules, sleep patterns, and lifestyle factors influencing circadian rhythms [4].

Objective outcomes of chronopharmacotherapy include improvements in attention, executive function, and daily functioning, alongside reductions in ADHD symptoms. Safety and exposure–response relationships are critical, as the therapeutic window for stimulants is narrow, and excessive exposure may lead to insomnia, anxiety, and cardiovascular issues [15,17]. Monitoring and adjusting dosages based on individual response and tolerability is essential [12].

Despite its promise, gaps remain in understanding and applying chronopharmacotherapy. More research is needed on its long-term effects on sleep and circadian health, and the development of personalized treatment algorithms incorporating genetic, environmental, and lifestyle factors. Further studies should explore combining chronopharmacotherapy with behavioral interventions, such as cognitive-behavioral therapy for insomnia (CBT-I), to enhance treatment efficacy [14,17,25].

### 3.3. The Human Layer: Implementation Coaching and Adherence in Adult ADHD

Low adherence is one of the most significant problems in the treatment of patients with chronic diseases, as it affects the effectiveness of the treatment process [27,28]. On one hand, some patients may struggle with building new habits, while on the other, they face the need to change long-established patterns of behavior to those more beneficial to their health. Regular medication intake, implementing the principles of chronotherapy, or increasing physical activity levels are just a few examples. Often, numerous changes are necessary, and maintaining an optimal level of adherence to therapeutic recommendations is an even greater challenge in the context of co-occurring ADHD. Moreover, when faced with an almost endlessly growing list of “tasks,” it is easy to lose motivation. Consequently, it is crucial to find an approach that helps patients not only to build a realistic plan but also to implement and sustain it.

The use of coaching in patients with ADHD may significantly increase the likelihood of adherence to therapeutic recommendations. This approach allows for a better-tailored plan for implementing changes, considering the patient’s capabilities and limitations in daily functioning. The benefits of implementing this form of support have been observed mainly in patients with chronic diseases, including increased physical activity [29], better glycemic control [30], and weight reduction in patients with obesity, with or without coexisting type 2 diabetes [31]. Coaching is typically used as an adjunct to standard treatment (inter alia: [32]), helping patients build a level of motivation that enables them to take the first step toward implementing medical recommendations.

Increasing attention is being paid to the potential benefits of using coaching in individuals diagnosed with ADHD (inter alia: [7,33,34,35]). Coaching goals often focus on areas such as time management, career planning, interests, or a healthy lifestyle [36]. Coaching is a promising form of support that can improve daily functioning [32,34], including academic [13,32] and occupational performance [7]. In a multiple case study involving adolescents, the use of cognitive-behavioral coaching (CBC) resulted in better coping with school responsibilities and increased self-esteem [37]. Most participants in that study were concurrently receiving pharmacological treatment [37], meaning that CBC coaching served as an adjunctive therapy.

A person conducting coaching should possess appropriate competencies, adhere to a Code of Ethics, and assess whether coaching is the most beneficial approach for a given individual (inter alia: [38]). This is intended to reduce the risk of adverse effects, which may arise not only from the coach’s insufficient preparation but also from the participant’s lack of readiness for the coaching process or the presence of mental health disorders requiring different interventions [38].

Coaching is not merely a subject of academic consideration or scientific research; it is an approach sought by some individuals with ADHD because it focuses on their existing resources, problem-solving, and facilitates better self-understanding [8,39]. The coaching process, and specifically the coach, is guided by what is important to the patient—reinforcing awareness of why achieving their chosen goal matters, the underlying values, and the consequences of inaction. During subsequent sessions, the patient creates their own action plan and develops strategies to cope with potential obstacles, fluctuating motivation, and delayed results. From a long and typically unenthusiastic to-do list, they select actions that are a priority at that moment.

Working through “micro-crises” is crucial—for instance, when a patient cannot fully adhere to their planned behavioral changes and begins to think, “I didn’t get up at the set time yesterday or the day before, so this whole chronotherapy thing isn’t for me.” This reflects “health-inhibiting thinking” [39]. Using cognitive-behavioral techniques in coaching [40], this thought can be reframed into a more constructive one: “The fact that I didn’t get up at the set time twice last week doesn’t mean that chronotherapy isn’t for me.”

This process is particularly important because most patients diagnosed with ADHD also struggle with sleep or circadian rhythm disorders, among which Delayed Sleep Phase Syndrome is predominant [41]. The consequences of this pattern were discussed earlier in the manuscript. There is a need for randomized controlled trials to verify the effectiveness of coaching in individuals with ADHD [42], especially in those with co-occurring sleep and circadian rhythm disturbances. A major challenge in evaluating this approach is the significant heterogeneity of coaching interventions, which often prevents comparison between studies (inter alia: [43]).

## 4. Discussion

### 4.1. Summary of Key Findings

This review explores the intricate relationship between methylphenidate, sleep, and the so-called “stimulant paradox” in adults with ADHD, proposing a conceptual framework that integrates chronopharmacotherapy and coaching. The stimulant paradox refers to the counterintuitive calming effect of stimulants like methylphenidate in individuals with ADHD, which contrasts with their stimulating effects in the general population [1]. Our analysis highlights how methylphenidate, when administered with consideration of individual circadian rhythms and sleep patterns, can optimize therapeutic outcomes while minimizing sleep disturbances [18,25]. Additionally, the role of coaching is examined as a complementary strategy to enhance adherence and manage the timing of medication intake, thereby supporting overall treatment efficacy.

### 4.2. Integration with Prior Work: Stimulant Paradox & Chronopharmacotherapy

The stimulant paradox remains a central theme in ADHD pharmacotherapy, with methylphenidate demonstrating efficacy in reducing core symptoms despite its classification as a stimulant [1]. This paradox is further complicated by the interplay between ADHD symptoms and disrupted sleep patterns, which are prevalent in this population [6]. Chronopharmacotherapy, which involves tailoring medication timing to align with an individual’s circadian and homeostatic sleep processes, offers a promising approach to address these complexities [4].

The two-process model of sleep regulation, comprising Process C (circadian) and Process S (homeostatic), provides a useful framework for understanding these interactions [20]. Process C governs the body’s internal clock, influencing sleep–wake cycles, while Process S reflects the accumulation of sleep pressure over time. Figure in this review illustrates how methylphenidate’s pharmacokinetics can be overlaid with these processes to optimize dosing schedules. By aligning medication administration with these biological rhythms, clinicians can potentially mitigate sleep-related side effects and enhance therapeutic efficacy [44].

### 4.3. Clinical Implications (Non-Prescriptive)

The integration of chronopharmacotherapy into clinical practice could offer significant benefits for adults with ADHD. By considering individual variations in circadian rhythms and sleep patterns, clinicians may better tailor treatment plans to optimize symptom control while minimizing adverse effects. This approach underscores the importance of personalized medicine, where treatment is adapted to the unique biological and lifestyle factors of each patient [3]. Additionally, incorporating coaching strategies can further support patients in managing their medication schedules and improving adherence, ultimately enhancing treatment outcomes.

### 4.4. Limitations of the Evidence and of This Review

While the conceptual framework proposed in this review is grounded in existing literature, several limitations must be acknowledged. The evidence base for chronopharmacotherapy in adult ADHD is still emerging, with much of the current research focusing on pediatric populations or lacking robust clinical trials [45]. Additionally, the heterogeneity of ADHD presentations and comorbidities poses challenges in generalizing findings across diverse patient groups. This review also relies on narrative synthesis, which, while valuable for integrating diverse sources, may introduce bias in the interpretation of findings.

### 4.5. Research Agenda: Design Priorities

Future research should prioritize well-designed clinical trials that explore the efficacy of chronopharmacotherapy in adult ADHD. These studies should aim to elucidate the optimal timing and dosing of methylphenidate in relation to individual circadian and homeostatic processes. Additionally, research should investigate the long-term effects of this approach on sleep quality and overall functioning. The development of standardized assessment tools to evaluate circadian rhythms and sleep patterns in ADHD populations would further enhance the precision of chronopharmacotherapy [6].

## 5. Conclusions

In conclusion, the integration of chronopharmacotherapy and coaching offers a promising framework for enhancing the management of adult ADHD. By aligning medication administration with individual circadian rhythms and supporting patients through coaching, clinicians can potentially improve treatment outcomes while minimizing adverse effects. This approach reflects a broader trend towards personalized medicine, emphasizing the need for continued research and collaboration across disciplines to refine and implement these strategies effectively.

## Data Availability

No new data were created or analyzed in this study.

## Abbreviations

The following abbreviations are used in this manuscript:

ADHD	Attention-Deficit/Hyperactivity Disorder
AI	Artificial intelligence
CBC	Cognitive-behavioral coaching
CBT-I	Cognitive-behavioral therapy for insomnia
CR	Controlled-release (formulation)
C_max_	Maximum (peak) plasma concentration
DLMO	Dim-light melatonin onset
ER	Extended-release (formulation)
IR	Immediate-release (formulation)
OROS	Osmotic-Release Oral System (formulation)
PD	Pharmacodynamics
PK	Pharmacokinetics
SCN	Suprachiasmatic nucleus
SOL	Sleep-onset latency
STEP	Sustaining Training Effects through Physical Activity
T_max_	Time to maximum plasma concentration
t_½_	Elimination half-life
